# Magnetic Resonance Imaging With a Novel Hip Flexion Scanning Position for Diagnosing Proximal Hamstring Tendinopathy

**DOI:** 10.1177/23259671241265130

**Published:** 2024-09-25

**Authors:** Aleksi Jokela, Pekka Niemi, Ilona Koski, Jussi Kosola, Xavier Valle, Ricard Pruna, Sakari Orava, Carles Pedret, Ramon Balius, Giulio Pasta, Juha-Jaakko Sinikumpu, Keijo Mäkelä, Lasse Lempainen

**Affiliations:** * University of Turku, Turku, Finland; † Department of Orthopaedics and Traumatology, Turku University Hospital, Turku, Finland; ‡ Hospital Mehiläinen Neo, Turku, Finland; § Hospital Pihlajalinna, Turku, Finland; ‖ Department of Orthopaedics and Traumatology, Kanta-Häme Central Hospital, Hämeenlinna, Finland; ¶ University of Helsinki, Helsinki, Finland; # Department of Orthopaedics and Traumatology, Hyvinkää Hospital, Hyvinkää, Finland; ** ICATME, Hospital Universitari Dexeus, UAB, Barcelona, Spain; †† Medical Department, FC Barcelona, Barcelona, Spain; ‡‡ Research Institute Orton, Helsinki, Finland; §§ Sports Medicine and Imaging Department, Clinica Diagonal, Barcelona, Spain; ‖‖ Catalan Sports Council, Government of Catalonia, Barcelona, Spain; ¶¶ Parma Calcio, Parma, Italy; ## Pediatric Orthopaedics and Traumatology, Oulu University Hospital, Oulu, Finland; a Clinical Medicine Research Unit, Medical Research Center MRC, University of Oulu, Oulu, Finland; b FinnOrthopaedics / Hospital Pihlajalinna, Turku, Finland; c Ripoll y De Prado, FIFA Medical Centre of Excellence, Madrid, Spain; d Department of Physical Activity and Health, Paavo Nurmi Centre, University of Turku, Turku, Finland; e Aspetar Orthopaedic and Sports Medicine Hospital, Doha, Qatar; Investigation performed at the Hospital Mehiläinen Neo, Turku, Finland

**Keywords:** proximal hamstring tendinopathy, magnetic resonance imaging, hamstring injuries, diagnostics

## Abstract

**Background:**

Making a diagnosis of proximal hamstring tendinopathy (PHT) may be challenging, as patients with correlating clinical symptoms may have normal or minimal findings on magnetic resonance imaging (MRI) scans.

**Purpose/Hypothesis:**

The purpose of this study was to assess the effect of a novel hip flexion (HF) scanning position on the MRI diagnosis of PHT. It was hypothesized that the HF position, which simulates the symptom-provoking sitting position, would reveal PHT pathology more accurately than the standard scanning position.

**Study Design:**

Cohort study (diagnosis); Level of evidence, 3.

**Methods:**

Patients with chronic PHT symptoms were included. Chronicity was defined as symptoms that were present for >3 months. Each patient underwent an MRI in 2 parts: (1) the standard pelvic examination in the supine position and (2) the novel HF position in which the patient lays on his or her side with the hip at 90° of flexion. Tendon insertion areas of the semimembranosus and the biceps femoris were analyzed independently by 2 experienced musculoskeletal radiologists, and the findings were classified as *normal*, *tendinosis*, or *rupture*. The MRI findings for both the standard and HF positions were compared in every patient, and the percentage of different diagnoses between the 2 MRI positions was reported.

**Results:**

In total, 38 patients (67 tendons) were analyzed. In 71% of the patients, the HF position revealed more severe injury than the standard position. The HF position showed a rupture in 16% of the tendons, with findings classified as tendinosis in the standard position. Of the tendons diagnosed as normal in the standard position, 6% were classified as rupture and 11% as tendinosis in the HF position.

**Conclusion:**

The novel HF scanning position offered additional value in the diagnosis of PHT in symptomatic patients when compared with the standard hip-in-neutral position. This position can improve the diagnostics of PHT, especially if an athlete or an active patient with gluteal area pain has normal or minimal MRI findings in the standard position.

Chronic hamstring overuse injuries and symptoms are common in athletes participating in sports such as long-distance running, sprinting, and hurdling.^4,11^ Proximal hamstring tendinopathy (PHT), also known as hamstring syndrome, is a chronic degenerative disease that is seen mostly in these athletically active patients and causes lower gluteal pain.^13,15^

The differential diagnosis and management of gluteal region pain is often challenging.^7,11^ The diagnosis of PHT is mainly based on patient's background, typical symptoms, clinical findings, and imaging findings.^13^ The symptoms at the lower gluteal area typically occur during running or while sitting for prolonged periods. Additionally, a patient with PHT may often be asymptomatic in a standing or lying position (hip in neutral position), whereas during hamstring elongation (eg, sitting, stretching, uphill running) the symptoms occur. Although the pain often appears without any sudden trauma, PHT can sometimes be preceded by previous acute hamstring injury or recurrent injuries.^10^

Magnetic resonance imaging (MRI) is typically used to diagnose hamstring injuries.^5,9^ The imaging findings guide decision-making concerning treatment by providing detailed information about the extent and severity of the injury.^10^ The typical MRI findings in PHT are swelling and/or tendon thickening at the insertion area of the hamstrings. Also, swelling and edema in the ischial tuberosity and a degenerative partial tendon rupture and/or longitudinal splits can be found at the insertion area.^5^ However, patients may have a typical symptomatology of PHT but imaging findings in MRI are minimal or even normal. This may lead to a delayed diagnosis and a prolonged time loss from sports.

In this study, we present a novel MRI scanning position that simulates sitting in the hip flexion (HF) position, which is a typical position causing symptoms in PHT, and demonstrate its feasibility for that purpose. The main aim of this study was to compare the novel HF scanning position with the standard position (neutral hip) for the MRI diagnosis of PHT. We hypothesized that the novel scanning position would be more sensitive than the standard position in patients with a presumed diagnosis of PHT as evidenced by clinical examination.

## Methods

The study protocol was approved by the ethics committee of the local hospital district. All included patients were physically active or recreational athletes. All patients were informed about the study setup, and they participated on a voluntary basis. The imaging studies were performed between February 2019 and January 2022.

### Study Participants

The study participants were prospectively recruited from the patients visiting a single orthopaedic surgeon (L.L.) due to chronic symptoms in the subgluteal area (one or both sides). Symptoms were classified as chronic if they had lasted >3 months. To be included, a patient had to have clinically diagnosed PHT with typical symptoms (pain in the lower gluteal area, especially during sitting, running, or stretching, that radiated down to the posterior thigh) lasting >3 months. Patients who met the inclusion criteria were informed about the study setup and protocol and were given a reasonable amount of time to consider whether they wished to participate. Patients incapable of going into the novel imaging position, those having previous hamstring injuries, and those having contraindications to MRI were excluded. Additionally, MRI had to be performed before possible therapeutic injections. Patients who met the inclusion criteria and gave their consent to participate were included.

### MRI Technique

All patients underwent MRI with a 3.0-T system (MAGNETOM Skyra; Siemens Healthineers) at the radiology unit of a private sports hospital. A combination of a 32-channel spine coil and an 18-channel flexible surface coil was used for signal reception.

**Figure 1.**
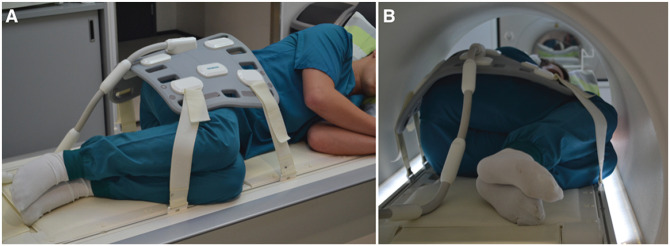
The novel magnetic resonance imaging scanning position of hip flexion (A) before and (B) during the scan in a patient who is 176 cm tall (body mass index, 23.9).

The MRI protocol consisted of 2 parts. In the first part, a standard pelvic examination with the patient supine and the hip in neutral position was performed. For the second part, the patients lay on their asymptomatic side with the symptomatic hip or both hips in flexion (Figure 1). The HF angle was aimed at 90°, which was estimated visually.

The standard part of the MRI protocol included the following turbo spin echo sequences: T2-weighted Dixon sequence in the coronal and sagittal planes, T1-weighted sequence in the coronal and axial planes, and T2- and intermediate-weighted sequences with fat suppression in the axial plane. The hip-in-flexion part included T2-weighted Dixon sequence in the coronal, axial, and sagittal planes. Parallel imaging (GeneRalized Autocalibrating Partial Parallel Acquisition [GRAPPA]) was utilized, with a factor of 2 to 3. Details of the sequence parameters are given in Table 1.

**Table 1:** Details of the MRI Sequence Parameters^*a*^

Sequence	Orientation	TR (ms)	TE (ms)	Slice Thk (mm)	In-Plane Res (mm)	FoV (mm)	FoV Phase (%)
Standard							
Dixon T2-w	Coronal	3500	57	4	0.6 × 0.6	320	118.80
T1-w	Coronal	700	11	3	0.7 × 0.7	370	100
T2-w fat sat	Axial	3430	52	3.5	0.7 × 0.7	340	68.80
T1-w	Axial	700	12	3.5	0.7 × 0.7	380	59.4
Intermediate-w	Axial	4080	41	3	0.6 × 0.6	320	100
Dixon T2-w	Sagittal	3050	62	3	0.7 × 0.7	340	71.9
Hip in flexion							
Dixon T2-w	Coronal	3500	57	4	0.6 × 0.6	320	118.80
Dixon T2-w	Axial	2800	57	3	0.6 × 0.6	320	118.80
Dixon T2-w	Sagittal	2600	60	3	0.8 × 0.8	340	100

a FoV, field of view; MRI, magnetic resonance imaging; Res, resolution; sat, saturated; TE, echo time; Thk, thickness; TR, repetition time; -w, weighted.

### MRI Interpretation

Two radiologists (P.N. and I.K.), with approximately 30 years of clinical experience in musculoskeletal MRI, independently and systematically assessed all MRIs for signs of PHT. The tendon insertion areas of the semimembranosus (SM) and the conjoint tendon of the biceps femoris (BF) and the semitendinosus were analyzed, and the findings were classified as *normal, tendinosis*, or *rupture* (the degenerative and long-term evolution disruptions of the tendon fibers). In terms of classifying tendinosis, tendon size and inhomogeneity generally correlate with a patient's symptoms.^5^ Although the best way to evaluate the thickening of the tendon would be to measure it, we found the resolution of the images to be not sufficient enough for reliable classification. Therefore, we used a 2-stage classification when assessing the thickening of the tendon, and inhomogeneity was visually evaluated. Tendinosis was further categorized into *slightly thickened* (homogeneous or inhomogeneous) or *clearly thickened* (homogeneous or inhomogeneous). Tendon ruptures were visually assessed and classified as *small* (less than half of the cross-sectional area ruptured), *wide* (more than half of the cross-sectional area ruptured), or *complete* (loss of the tendon continuity).

After the initial assessment, a meeting was held between the radiologists, and any discrepancies were discussed until a consensus was reached. In addition, the actual flexion angles were determined by measuring the angle between the femur and the long axis of the ischial tuberosity on MRI.

The MRI findings from the HF and standard positions were compared in every patient. The results were reported as the percentage of different diagnoses between the 2 MRI positions. In addition, the results of the 2 positions were compared to evaluate the diagnostic value of the HF position.

## Results

A total of 38 patients with typical PHT symptoms (67 thighs scanned) were included in this study. The study group included 10 male and 28 female patients (mean age, 37.5 years; range, 14-58 years). The mean HF angle in the HF position was 54° (range, 28°-81°).

### MRI Findings

Details of the MRI findings are presented in Table 2. In the standard position, the SM tendon was classified as normal in 39% (n = 26/67), tendinosis in 28% (n = 19/67), and rupture in 33% (n = 22/67). The BF tendon was classified as normal in 15% (n = 10/67), tendinosis in 54% (n = 36/67), and rupture in 31% (n = 21/67). In the HF position, the SM tendon was normal in 30% (n = 20/67), tendinosis in 28% (n = 19/67), and rupture in 42% (n = 28/67), and the BF tendon was found to be normal in 15% (n = 10/67), tendinosis in 48% (n = 32/67), and rupture in 37% (n = 25/67).

**Table 2:** Magnetic Resonance Imaging Findings in the Standard and Hip Flexion Positions (N = 67 Thighs)

Diagnosis	Standard Position	Hip Flexion Position
	n (%)	n (%)
Semimembranosus Tendon		
Normal	26 (39)	20 (30)
Tendinosis	19 (28)	19 (28)
Slightly thickened		
Homogeneous	11 (16)	8 (12)
Inhomogeneous	6 (9)	7 (10)
Clearly thickened		
Homogeneous	0 (0)	3 (4)
Inhomogeneous	2 (3)	1 (1)
Rupture type	22 (33)	28 (42)
Small	18 (27)	13 (19)
Wide	4 (6)	13 (19)
Complete	0 (0)	2 (3)
Biceps Femoris Tendon		
Normal	10 (15)	10 (15)
Tendinosis	36 (54)	32 (48)
Slightly thickened		
Homogeneous	20 (30)	14 (21)
Inhomogeneous	12 (18)	9 (13)
Clearly thickened		
Homogeneous	1 (1)	5 (7)
Inhomogeneous	3 (4)	4 (6)
Rupture type	21 (31)	25 (37)
Small	19 (28)	11 (16)
Wide	2 (3)	14 (21)
Complete	0 (0)	0 (0)

Compared with the standard position, the HF position revealed more severe injury in 71% (n = 27/38) of the patients. The HF position revealed a rupture in 16% (n = 9/55) of the tendons diagnosed as tendinosis in the standard position. In 6% (n = 2/36) of the tendons classified as normal in the standard position, a rupture was classified in the HF position. Additionally, in 11% (n = 4/36) of the tendons diagnosed as normal in the standard position, the HF position showed a tendinosis. These results are presented in Table 3.

**Table 3:** The Diagnostic Value of the HF Position Compared With Standard Position^*a*^

Comparison	n/Total (%)
HF position revealed more severe injury	
All patients	27/38 (71)
Semimembranosus tendon	26/67 (39)
Biceps femoris tendon	21/67 (31)
HF position revealed a rupture, although	
Standard position showed tendinosis	9/55 (16)
Standard position showed normal findings	2/36 (6)
HF position revealed a tendinosis, although standard position showed normal findings	4/36 (11)

a HF, hip flexion.

### MRI Examples

**Figure 2.**
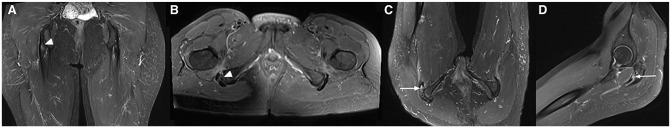
T2-weighted Dixon magnetic resonance imaging scans from a 55-year-old woman with hamstring symptoms on both sides. Standard (A) coronal and (B) axial images show tendinosis of the proximal tendons on the right side (arrowheads). With the hip flexion position, a partial tear of the semimembranosus tendon is detected, as seen on (C) axial and (D) sagittal images (arrows).

**Figure 3.**
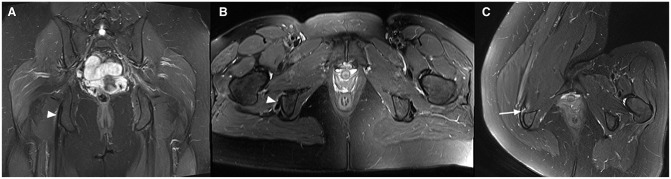
T2-weighted Dixon magnetic resonance imaging scans from a 38-year-old woman with symptoms on the right side. Standard (A) coronal and (B) axial images show mild edema around the proximal hamstring tendons (arrowheads), but the tendons appear quite normal. (C) An axial image with right hip flexion reveals a partial rupture of the semimembranosus tendon (arrow).

**Figure 4.**
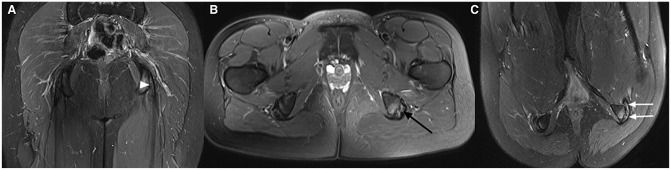
T2-weighted Dixon magnetic resonance imaging scans from a 25-year-old woman with hamstring symptoms on the left side. (A) Standard coronal image shows inhomogeneous hamstring tendons (arrowhead). (B) Standard axial image also shows some irregularity on the medial surface of the tendons (black arrow). (C) An axial image with hip flexion uncovers a quite large tear of the proximal hamstring tendons (white arrows).

**Figure 5.**
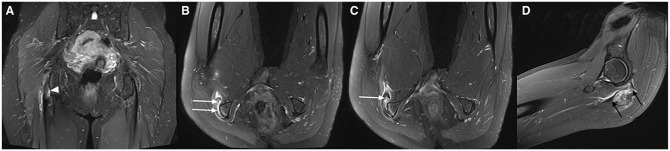
T2-weighted Dixon magnetic resonance imaging scans from a 57-year-old man with hamstring symptoms on the right side. (A) A standard coronal image shows a partial tear of the proximal semimembranosus tendon (arrowhead). (B and C) Axial images with hip flexion reveal that the tear is much larger, as almost a complete detachment of the tendon is seen (white arrows). (D) The total length of the rupture is also portrayed on a sagittal image with hip flexion (between black arrows).

To demonstrate the findings of the novel scanning position, MRI examples from patients included in this study are presented in Figures 2 to 5.

## Discussion

The main finding of this study was that patients undergoing MRI in a novel HF scanning position offered additional value in diagnosing PHT when compared with the standard position. In 71% of the patients, the HF position revealed more severe injury than the standard position. The HF position revealed a rupture in 16% of the cases with tendinosis and 6% of the cases with normal findings in the standard position.

Lempainen et al^11^ previously investigated the histopathological findings of PHT. The authors took biopsy samples from 15 of the 103 operated tendons for analysis by a pathologist. In that study, the control biopsy specimen showed no signs of tendinosis, whereas the typical histologic findings of tendinosis were evident in all other biopsy specimens. This finding is similar to that of Bäcker et al^1^ concerning the Achilles tendon, in which all ruptured tendons needing surgical treatment were previously degenerated to some degree. In the study of Lempainen et al,^11^ the typical morphologic findings of tendinosis (rounding of tenocyte nuclei, increased ground substance, collagen disintegration, and increased vascular proliferation) were found in various degrees. In MRI, a thickened SM tendon was found in all cases before surgery.

The differential diagnosis of gluteal region pain disorders in general is challenging. Differential diagnostics of PHT include conditions such as piriformis syndrome, where the sciatic nerve is compressed by the piriformis muscle, and often causes buttock pain, hip pain, or sciatica.^14^ Similar to PHT, the pain is commonly aggravated by prolonged sitting, such as driving a car, or by rising from a seated position.^14^ Additionally, radicular symptoms of sciatica can mimic the typical clinical picture of PHT, as the pain usually radiates distally from the buttock when the hip is flexed.^16^ Obviously, various conditions can cause the entrapment of the sciatic nerve and cause pain to the posterior hip and thigh. In a systematic review published in 2020, Kizaki et al^8^ defined deep gluteal syndrome by 3 characteristics: nondiscogenic, sciatic nerve disorder, and nerve entrapment in the deep gluteal space. However, they explained that the term “deep gluteal syndrome” potentially covers cases with buttock pain in the deep gluteal space caused also by other factors than sciatic nerve entrapment, such as PHT.^8^ Also, in PHT, adhesions between sciatic nerve and proximal hamstring tendons may sometimes be present, and furthermore in some cases the sciatic nerve may be compressed by the thickened tendons.^10^ Due to the complex differential diagnostics and challenging management of gluteal pain, the development of new imaging modalities is important. Patients with typical PHT symptoms should be carefully assessed based on patient's background, symptoms, and clinical and imaging findings.

MRI and ultrasound are the imaging modalities of choice for diagnosing PHT. MRI is more sensitive due to its better soft tissue contrast, while ultrasound is a cost-effective and easily available alternative.^5,9^ The objective of imaging is to confirm the pathology of hamstring tendon and evaluate the severity of the injury.^10^ The imaging results may be helpful in assessing which patients may benefit from surgical treatment and how long rehabilitation period can be expected. The typical findings of PHT on MRI are increased signal intensity on T1- and T2-weighted images, thickening of the tendons, peritendinous edema, and bone marrow edema–like changes in the ischial tuberosity.^19^ However, it has been found that signal changes on T1- and T2-weighted images are commonly present also in asymptomatic patients.^5^ The pathological MRI findings in the proximal hamstring tendon among asymptomatic patients may be due to the adaptation process that is the reaction of the tendons to an increased loading. It has been discussed that peritendinous edema with a distal feathery pattern, bone marrow edema–like changes, and thickening of tendons are more reliable signs of tendinopathy in MRI.^10^ However, all these changes can also be found in asymptomatic patients, and therefore, correlation between imaging and clinical symptoms is the most important aspect in diagnosis of PHT.^10^ On the other hand, MRI findings may be normal or minimal in patients having difficult symptoms, or the primary suspected diagnosis can be wrong, as we found in this study. A recently published study showed that 8 of 31 symptomatic patients diagnosed with PHT had normal MRI findings, suggesting that a quarter of the symptomatic patients had no tendinopathy findings in MRI.^3^ This highlights the complexity of the diagnostics of PHT, indicating a considerable need for better tools and protocols in the diagnostics of PHT. The novel scanning position presented in this study offers a potential tool to develop the diagnostics of PHT.

From an imaging perspective, the main advantage of the novel approach is that it allows a better view to lower parts of the tendon attachments. It is often the case in the standard scanning position that small tears are not depicted because the injured tendon stays close to the bone. Therefore, the fact that the changes in proximal hamstring tendons can be seen more clearly with the novel scanning position is due to mechanical factors. As the muscle group is more stretched in the HF position, the structural changes and injuries can be better visualized. We hypothesized that this is the explanation why the novel position of scanning PHT seems to be more sensitive than the standard position. Additionally, we found that in the HF position, the direction of the tendons is more perpendicular to the footprint of the origin, the proximal parts of the tendons become flatter and more deltoid shaped, and the BF tendon turns from its posterior position to cover the SM tendon. These findings may also explain the better visibility of the tendons in the HF position.

From a clinical perspective, the main goal of this study was to enable the early and accurate diagnostics of PHT to prevent future ruptures and symptoms. Since the diagnosis has been made, the choice of management approach is crucial to reach a good outcome. Management protocols for PHT mainly focus on conservative treatment with the goal of reducing symptoms and maintaining physical function.^13^ Nonsurgical interventions include strength and mobility exercises, corticosteroid injection, platelet-rich plasma injection, shockwave therapy, and high-power laser therapy.^7,10,13,17^ In this sense, better knowledge of the exact location of the affected insertional zone can be of great help when performing an ultrasound-guided infiltration or the most exact application of shockwave therapy, and high-power laser therapy. However, there is lack of high-level evidence on the effect of different interventions on PHT.^12^ In recalcitrant cases with unsatisfying outcome despite the appropriate and long enough (>3-6 months) conservative treatment, surgical treatment has been found to result in good outcome in management of PHT.^2,6,11,13,18^ However, according to a recently published systematic review of PHT interventions by Nasser et al,^12^ there is insufficient scientific evidence to prove any intervention superior to another due to the lack of comparative studies. This study may help to diagnose PHT earlier, which can lead to better outcomes in rehabilitation.

### Limitations

This study has certain limitations. First, the image analysis was not performed in a completely blinded manner, as the radiologists knew that hamstring-related problems were suspected; therefore, it is evident that the comparison between the findings on standard and hip-in-flexion images is susceptible to unjust interpretation. However, this is often the case when new imaging protocols are introduced. Additionally, since this study reported a completely new imaging protocol that could offer beneficial additional information, we did not perform observer reliability testing, which is a clear limitation. Further research is needed to assess intra- and interobserver reliability of our protocol.

There is also a learning curve: Once the experience of hip-in-flexion images is acquired, there is a difference in the way standard images are evaluated. Our goal was to image the hips at 90° of flexion. However, the measured mean of flexion angle was only 54° in reference to the ischial tuberosity. The possible explanations are pelvic anteversion and the difference between patients’ mobility. It has been found that asymptomatic patients may also have tendinopathy findings in MRI.^3,5^ However, more often, the typical PHT findings in asymptomatic patients were associated with older age, likely relating to natural degenerative changes.^3^ A larger study including an age-adjusted control group would be needed to establish how well our findings correlate with the symptoms of the study participants.

Also, when assessing tendinopathy on MRI, the possibility of magic angle artifacts should be taken into consideration, especially when the direction of the tendons is altered in reference to the main magnetic field. However, we believe that this did not affect our results. Finally, the physical examination findings of other gluteal pain conditions can mimic PHT, so assuming PHT based on clinical exam alone is a limitation.

## Conclusion

The novel MRI scanning position of the patient imaged with the hip flexed offered additional value in the diagnosis of PHT in symptomatic patients, when compared with the standard hip-in-neutral position. Therefore, we recommend this additional position be included, especially if an athlete or an active patient with gluteal area pain has normal or minimal MRI findings in the standard scanning position.
